# SquiDBase: a community resource of raw nanopore data from microbes

**DOI:** 10.1093/nargab/lqaf213

**Published:** 2026-01-08

**Authors:** Wim L Cuypers, Halil Ceylan, Eline Turcksin, Laura Raes, Nicky de Vrij, Johan Michiels, Sandra Coppens, Tessa de Block, Daan Jansen, Kevin K Ariën, Philippe Selhorst, Koen Vercauteren, Julia M Gauglitz, Wout Bittremieux, Kris Laukens

**Affiliations:** Adrem Data Lab, Department of Computer Science, University of Antwerp, 2000 Antwerp, Belgium; Adrem Data Lab, Department of Computer Science, University of Antwerp, 2000 Antwerp, Belgium; Adrem Data Lab, Department of Computer Science, University of Antwerp, 2000 Antwerp, Belgium; Adrem Data Lab, Department of Computer Science, University of Antwerp, 2000 Antwerp, Belgium; Adrem Data Lab, Department of Computer Science, University of Antwerp, 2000 Antwerp, Belgium; Clinical Immunology Unit, Department of Clinical Sciences, Institute of Tropical Medicine Antwerp, 2000 Antwerp, Belgium; Virology Unit, Department of Biomedical Sciences, Institute of Tropical Medicine Antwerp, 2000 Antwerp, Belgium; Virology Unit, Department of Biomedical Sciences, Institute of Tropical Medicine Antwerp, 2000 Antwerp, Belgium; Clinical Virology Unit, Department of Clinical Sciences, Institute of Tropical Medicine Antwerp, 2000 Antwerp, Belgium; Clinical Virology Unit, Department of Clinical Sciences, Institute of Tropical Medicine Antwerp, 2000 Antwerp, Belgium; Virology Unit, Department of Biomedical Sciences, Institute of Tropical Medicine Antwerp, 2000 Antwerp, Belgium; Virology Unit, Department of Biomedical Sciences, Institute of Tropical Medicine Antwerp, 2000 Antwerp, Belgium; Clinical Virology Unit, Department of Clinical Sciences, Institute of Tropical Medicine Antwerp, 2000 Antwerp, Belgium; Adrem Data Lab, Department of Computer Science, University of Antwerp, 2000 Antwerp, Belgium; Adrem Data Lab, Department of Computer Science, University of Antwerp, 2000 Antwerp, Belgium; Adrem Data Lab, Department of Computer Science, University of Antwerp, 2000 Antwerp, Belgium

## Abstract

Nucleotide sequences in the FASTQ or BAM format are widely shared, yet derived from platform-specific raw data outputs that differ across sequencing platforms. In Oxford Nanopore Technologies (ONT) sequencing, raw signal data contain valuable biological information and enable basecaller optimization and modification detection. These raw signals also underpin algorithms that could improve ONT device portability and enhance target enrichment efficiency through adaptive sampling. Nevertheless, the storage and sharing of raw nanopore data remain limited due to technical constraints and the lack of standardized and centralized infrastructure. To address this challenge, we developed SquiDBase (https://squidbase.org), a dedicated repository for raw microbial nanopore sequencing data with linked processed data and metadata. To maximize immediate utility, we built SquiDPipe, a Nextflow pipeline for the automated removal of human reads from raw nanopore data, sequenced 24 clinically relevant viruses and incorporated them into SquiDBase, and added publicly available reference datasets and new community contributions. By offering a centralized, open-access raw data collection platform, SquiDBase facilitates data sharing, enhances reproducibility, and supports the development and benchmarking of computational tools, reinforcing open science in nanopore sequencing.

## Graphical Abstract

**Graphical Abstract:**
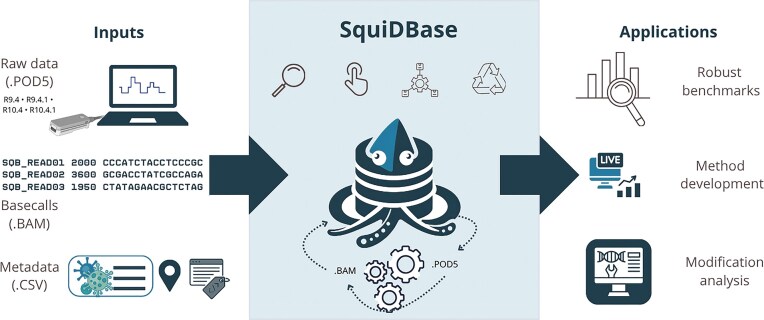

## Introduction

Raw data—the unmodified output of experimental instruments—serves as the foundational record of scientific observations, while downstream data processing may introduce bias or information loss [1, 2]. In nucleic acid sequencing, raw reads are typically shared as FASTQ or unaligned BAM files through public repositories [3, 4]. Generated through “basecalling” [5], these files contain nucleotide sequences and quality scores, with unaligned BAM carrying additional structured metadata [3, 6]. Because the initial raw data, its utility, and long-term storage needs vary significantly across sequencing platforms, it is worth considering whether sharing minimally processed, instrument-native raw signal could further advance open science and reproducibility.

In fluorescence-based sequencing-by-synthesis technologies (e.g. Illumina and PacBio), nucleotide incorporation signals are captured and automatically processed into high-quality FASTQ files [7], reducing the need to handle or store raw data. While these methods provide highly accurate sequences, extracting additional biological insights (e.g. DNA methylation) requires specialized preprocessing (e.g. bisulfite conversion) or additional kinetic information, as seen in PacBio’s HiFi sequencing [8]. In contrast, nanopore sequencers detect changes in ionic current in real-time as nucleic acids pass through a nanopore, recording their native state [9, 10], which creates multiple incentives for retaining and analyzing raw data. First, basecaller accuracy on native DNA improves when training data includes strain-specific epigenetic modifications [11–13]. Second, raw data support downstream analyses, including the detection of splice junctions [14], tandem repeats [15–17], and base modifications [18, 19], which in turn enable classification models [20] and *de novo* motif discovery [21]. Third, advancements in real-time analysis, including adaptive sampling and increased device portability, have intensified the search for efficient raw signal processing that circumvents graphics processing unit-intensive basecalling. Proof-of-concept tools include UNCALLED [22], RawHash2 [23, 24], Sigmoni [25], SigMap [26], BaseLess [27], and SquiggleNet [28]. However, their development and benchmarking remain constrained by the limited availability of high-quality raw data from diverse species.

Despite the clear benefits of preserving raw nanopore data for algorithm development, re-analysis, meta-analyses, and benchmarking, they are rarely shared in the public domain [29, 30]. Existing options include uploading to SRA/ENA as .tar archives, but this is inconvenient for reuse and discovery and lacks harmonized nanopore-specific metadata and explicit links to derived outputs. Consequently, many users upload only processed data in the FASTQ format, leading to data loss. To overcome these challenges and ensure long-term accessibility of raw nanopore data in accordance with open-science principles, we developed SquiDBase (short for “Squiggle database”), a repository designed for storing and sharing raw nanopore signals from microbial sequencing projects (encompassing prokaryotes, microbial eukaryotes, and viruses) with corresponding basecalled data. Our focus on microbial data stems from their scalability, reduced privacy constraints compared to human genomics, and their critical role in pandemic preparedness. This emphasis also fills an important gap, as current large-scale nanopore sequencing efforts prioritize human genomics, leaving microbial datasets, for instance those needed for benchmarking taxonomic classifiers [31], relatively scarce. By centralizing these data, SquiDBase significantly enhances the accessibility and reusability of raw microbial nanopore data, facilitating experimental research and the development and benchmarking of new algorithms.

## Materials and methods

SquiDBase (https://SquiDBase.org) is a user-friendly repository to store raw nanopore data in the POD5 file format, along with basecalled data and associated metadata, with a focus on microbial datasets relevant to infectious disease and metagenomics research.

### Database structure and implementation

SquiDBase stores all metadata linked to a set of raw nanopore reads in a PostgreSQL database, structured to adhere to third normal form (3NF). This design minimizes redundancy and ensures data integrity by organizing tables so that all non-key attributes depend solely on primary keys. We employ Alembic for database migrations, enabling us to track changes and maintain version control over the database schema. This ensures consistent application of updates and modifications across environments.

In total, 11 tables contain all information linked to a given submission in SquiDBase (Supplementary Table S1 and  Supplementary Fig. S1). These include tables for user data (*users*), technical details associated with the nanopore sequencing run (*pod5_read, nanopore_kit*), metadata related to the sample that was sequenced (*pod5_file, country, source, ncbi_taxon, diagnostic*), and a table for managing submissions (*submissions*).

### Data upload and standardization

To contribute to SquiDBase, users must create an account before they can upload data. Facilitating a secure, efficient, and responsive user experience, SquiDBase incorporates a registration and authentication system backend powered by FastAPI, while the frontend leverages Nuxt.js. User sessions are maintained using JSON web tokens, which securely transmit information between parties and authenticate users, keeping their sessions active without compromising security.

Once registered, raw nanopore data in the POD5 format can be uploaded along with basecalled data in the BAM format and comprehensive metadata in a predefined CSV format to enhance the utility of the dataset. Metadata fields include the filename, the taxonomic identifier of the predominant species in the sample, fields that comprehensively describe the sequencing library preparation as these factors influence the raw signal, and additional metadata fields detailed in Table 1. Where applicable, we enhance standardization by using established ontologies and controlled vocabularies. For example, we employ UBERON [32], ENVO [33], and FOODON [34] to specify the sample source and the OBI ontology [35] to describe the diagnostic method. Additionally, we use NCBI Taxonomy [36] to classify biological organisms and ISO 3166 two-letter country codes to indicate the country of origin and isolation (Table 1).

**Table 1.** Metadata fields in SquiDBase accompanying each upload

Field/variable name	Description	Data type	Example
Filename	The file name of the POD5 sequencing data to be uploaded	String	37124_CHIKV-1.pod5
Species TaxID	NCBI taxonomy identifier for the microbial species in the sample	NCBI taxonomy	37124
Year of isolation	The year the pathogen was collected or isolated from the host. For non-pathogenic organisms and metagenomic samples, this refers to the year the sample was collected	Integer	2014
Country of isolation	The country where the organism was isolated or the sample was collected	ISO 3166 country code	BE
Geographic origin	The likely country of origin of the organism or infection, if known. This may differ from the country of isolation—for example, in travel-related infections. For pathogens, this refers to the most probable geographic source of the infection	ISO 3166 country code	ET
Strain or lineage	The specific strain, lineage, or sequence type of the uploaded pathogen data	String	BA.5
Source ID	A unique identifier for the source of the pathogen sample, utilizing the UBERON, ENVO, or FOODON ontologies	UBERON, ENVO, and FOODON ontologies	UBERON:0000178
Host TaxID	NCBI taxonomy identifier for the host species from which the pathogen was isolated, if applicable	NCBI taxonomy	9606
Internal lab ID	The internal identification code assigned to the sample by the laboratory	String	PLAS-ETH-2023–0147
Diagnostic method	The diagnostic method used to detect the pathogen, if applicable, is represented using the OBI ontology	OBI ontology	OBI:0 003 045
Library source	Origin of the nucleic acid material	String	Genomic
DNA input type	Method used to select or amplify nucleic acid molecules prior to sequencing.	String	Random
Target scope	Intended sequencing strategy or scope of the assay	String	WGS
Remarks	Additional notes or remarks about the sample, often for internal collection records	Text field	“Strain donated by institute X.”, or “Collected for the SquiDBase project.”

Standardized ontologies and conventions were used where possible, such as UBERON for source classification and ISO 3166 for two-letter country codes. The table includes the field name, a description, the data type, and an example for each metadata field.

Each dataset includes an information field that supports free-text input, allowing users to provide essential details about the data. This field is intended for documenting the rationale of the study, a brief methodological summary, the source publication (preprint or article), and notes on any bioinformatics preprocessing. It is the only post-submission field that contributors can edit, for example, to add the final publication link or DOI once available.

During upload, SquiDBase automatically extracts and stores comprehensive sequencing run metadata from submitted POD5 files, including the type of experiment, device model, flow cell specifications, used library preparation kit, acquisition parameters, and real-time throughput (Table 2).

**Table 2.** Information on the sequencing run that is extracted from POD5 files and stored in a separate “pod5_file” table and other tables as needed to maintain third normal form (3NF) within SquiDBase

Field from POD5 file	Description	Example
context_tags.experiment_type	Type of experiment conducted	genomic_dna
sample_rate	Sequencing run sample rate in samples per second	4000
context_tags.selected_speed_bases_per_second	Approximate number of bases read per second during sequencing	400
sequencer_position_type	Model of sequencing device used	MinION Mk1B
sequencing_kit	Sequencing kit used for library preparation	sqk-lsk114
flow_cell_product_code	Product code indicating the flow cell type	FLO-MIN114

### Preprocessing pipeline for data preparation and human read removal

To facilitate standardized POD5 file processing and metadata formatting for SquiDBase submissions, we developed SquiDPipe, a Nextflow-based workflow (https://github.com/SquiDBase/SquiDPipe). SquiDBase currently operates under the assumption that all reads within a POD5 file share identical metadata, and accordingly, SquiDPipe is optimized for three common submission types: (i) pure isolates, (ii) metagenomes (with appropriate NCBI metagenome-level taxonomic identifiers), and (iii) clinical samples filtered to exclude human-derived reads. In isolate mode, applicable to the first two categories, SquiDPipe partitions reads by barcode only, splitting POD5 files using FASTQ read–ID linkage without any downstream classification or mapping. This enables straightforward generation of partitioned and renamed POD5 files and metadata compatible with SquiDBase. In contrast, the taxonomy-guided subset mode, intended to purge clinical samples of human reads, employs an initial classification step from basecalled read files followed by alignment to a curated reference panel (RefSeq prioritized, with GenBank fallback and optional decoy sequences such as human). Reads supporting the selected taxonomic rank (species-level by default, but user-configurable) are retained, and their read IDs are used to extract corresponding signal data from the POD5 file into species-specific subsets. The output comprises POD5 files enriched for individual target species and summary mapping statistics across all identified taxa. For any type of submission, if full metadata are provided as input, SquiDPipe also generates a SquiDBase-compatible CSV, streamlining final submission. Further implementation details are provided in Supplementary Methods Section S1.

### Data retrieval and access

Once uploaded, raw nanopore data are accessible to the scientific community for reanalysis and further research. Users can search for datasets via the “Browse Datasets” tab (accessible at https://squidbase.org/submissions) (Supplementary Fig. S2). To facilitate user exploration, the page includes a filtering feature that allows users to filter datasets based on the nanopore flow cell type (e.g. R9.4.1 or R10.4.1). This functionality is particularly valuable as the flow cell type is a critical factor influencing raw signal characteristics, which is essential for developing and optimizing analytical algorithms.

Users can access each dataset through a unique SquiDBase identifier (*e.g*. https://squidbase.org/submissions/SQB000004). Each dataset page includes a brief text summary, a visual overview of the metadata, and a section for downloading the data directly from the browser (Supplementary Fig. S3). A query functionality further enables users to search for files based on specific metadata (e.g. taxonomic identifier) within a submission. Users can access full metadata for any file by clicking an eye icon, which also provides an MD5 checksum for file integrity verification (Supplementary Fig. S4).

SquiDBase employs presigned URLs for secure file transfers, which expire after 7 days to prevent unauthorized access or hotlinking. Users access files via the submission page, maintaining dataset context. JSON-RPC facilitates smooth communication between the Nuxt.js frontend and the FastAPI backend, ensuring efficient data exchange for user authentication and core functionalities. Batch downloading of data for an entire submission is facilitated through scripts, accessible directly from the relevant pages. These scripts contain pre-configured curl commands for POD5 files and the corresponding BAM file that also remain valid for up to 7 days.

Expansion and maintenance of the system are further facilitated by FastAPI’s built-in support for OpenAPI, which provides comprehensive API documentation. This feature simplifies the integration of new frontends, such as Python libraries or command-line tools, in the future. Developers can easily access and understand the available endpoints, ensuring that the system remains adaptable and scalable as new features or tools are introduced.

### Legacy R9 data and expanding public R10.4.1 collections

To support benchmarking, we centralized legacy datasets generated using the older R9 flowcells from sources like SRA and CADDE on AWS into SquiDBase. These datasets have been previously used for benchmarking tools such as RawHash, Sigmoni, and others [22, 24, 25, 28]. The collection includes R9 datasets for *Escherichia coli*, SARS-CoV-2, and *Chlamydomonas reinhardtii* and were converted from FAST5 to POD5 using the POD5 package v0.3.15 (https://github.com/nanoporetech/pod5-file-format) and merged into larger files.

Publicly available R10.4.1 sequencing data for microbes remain limited and scattered. To address this gap, and given the significance of viruses in pandemic preparedness, we prioritized sequencing 20 clinically relevant viral species (68 isolates). Included in SquiDBase were Chikungunya virus (CHIKV-1, CHIKV-2, CHIKV-3, CHIKV-4), Eastern Equine Encephalitis virus (EEEV), Dengue virus (DENV1, DENV2, DENV3, DENV4), Human Immunodeficiency virus (HIV), Japanese Encephalitis virus (JEV), Mayaro virus (MAYV), Monkeypox virus (MPOX), O’nyong-nyong virus (ONNV), Rift Valley Fever virus (RVFV), Sandfly Fever Naples virus (SFNV), Sandfly Fever Turkey virus (SFTV), Sindbis virus (SINV), Severe Acute Respiratory Syndrome Coronavirus 2 (SARS-CoV-2), Tick-borne Encephalitis virus (TBEV), Usutu virus (USUV), Venezuelan equine encephalitis virus (VEEV), West Nile virus (WNV), Western Equine Encephalitis virus (WEEV), Yellow Fever virus (YFV), and Zika virus (ZIKV). In addition, we added data originating from *Plasmodium falciparum*, which is responsible for the majority of malaria cases and frequently harbors drug resistance mutations. Wet-lab procedures, sequencing, and bioinformatics processing are described in Supplementary Methods Section S2.

Beyond the viral and *Plasmodium falciparum* datasets we generated, SquiDBase also incorporates additional R10.4.1 sequencing data contributed by the wider research community. These include both publicly available datasets and previously unpublished datasets shared by collaborators interested in expanding the utility of SquiDBase. The SquiDBase landing page provides regularly updated statistics and offers a searchable interface that summarizes dataset sizes across taxonomic levels, including superkingdom, phylum, order, family, and species.

### Evaluation of raw signal matching accuracy using SquiDBase datasets

To demonstrate the utility of SquiDBase, we evaluated the raw signal–matching tool RawHash2 on real-world nanopore datasets obtained via the bulk download feature in SquiDBase. Our test set comprised five datasets: *E. coli* R9.4.1 and R10.4 previously used in RawHash2 benchmarking, plus new-to-benchmarking data for *Salmonella enterica* R9.4.1 and R10.4.1, and *E. coli* R10.4.1, originating from clinical isolates (Supplementary Table S4). Dataset details, tool configurations, and evaluation criteria are provided in Supplementary Methods Section S3.

## Results and discussion

We developed SquiDBase to promote the sharing of raw nanopore data in POD5 format originating from microbial sources by ensuring they are findable, accessible, interoperable, and reusable (FAIR) [37].

SquiDBase provides a streamlined process for researchers to share and access raw nanopore data, even without prior bioinformatics expertise. Data can be uploaded using a unique SquiDBase identifier, allowing the community to build upon existing data generation efforts and maintain consistent benchmarks over time. Submission methods vary by sample type: single-specimen or single-species datasets can be uploaded directly, while clinical samples containing human and microbial reads require preprocessing with the SquiDPipe pipeline, which we developed to remove human data. Metagenomic samples composed of multiple non-human species must include the appropriate NCBI taxonomic identifier for accurate classification. Once deposited, datasets remain openly accessible via the browse page or direct submission URL (e.g. https://squidbase.org/submissions/SQB000004) without login requirements. Users are able to retrieve raw nanopore data in POD5 format and basecalled data in the BAM format. To ensure that the paired raw and basecalled datasets remain aligned with ongoing improvements in basecalling, SquiDBase treats all submissions as “living data”. We aim to re-basecall all POD5 reads on a quarterly basis and release updated, versioned BAM files accordingly. Comprehensive documentation and our preprocessing tool SquiDPipe further enhance the utility of SquiDBase, making it a robust resource for the research community.

To maintain compatibility with prior benchmarking efforts, we have integrated datasets generated with R9 flow cell types, which were previously dispersed across multiple sources. These datasets are now centralized and accessible from the dataset overview page. Given the limited availability of recent R10.4.1 data, we have prioritized expanding the collection of publicly available raw nanopore data. This focused on viral datasets, contributing data for 68 viral isolates, encompassing 20 clinically relevant viral species. Descriptions on sequencing depth and coverage for each of the viral isolates sequenced for this project can be found in Supplementary Table S2. Furthermore, we have contributed raw sequencing data of *Plasmodium falciparum*, the predominant malaria-causing species. Data from four samples derived from three human patients were included. Chromosome-wide coverage statistics for these *P. falciparum* samples are provided in Supplementary Table S3. Thanks to further public datasets and contributions from the broader research community, SquiDBase currently already hosts over 1200 gigabytes of raw nanopore data from 39 distinct species. Ongoing community submissions are expected to further extend the coverage of SquiDBase.

**Figure 1.**
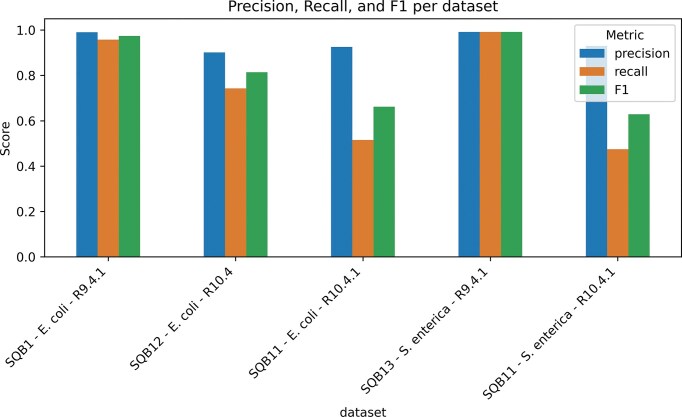
SquiDBase datasets enable real-world evaluation of raw signal alignment tools. Precision, recall, and F1 score for RawHash2 are shown per dataset, computed from read-level assignments against curated references. Labels on the *x*-axis refer to the SquiDBase entry (e.g. SQB1 = https://squidbase.org/submissions/SQB000001), followed by the name of the species in the dataset and the type of flowcell used to generate the data. Further information on the datasets can be found in Supplementary Table S4.

As a use case, we leveraged SquiDBase to evaluate the raw signal mapping tool RawHash2 across taxa and ONT chemistries. Consistent with prior benchmarking [38], RawHash2 performed best on the legacy R9 datasets, maintaining high precision and recall even on an unseen clinical isolate. Performance declined on datasets generated with the more recent R10.4 flow cell and was lowest for data generated by the currently supported R10.4.1 flow cell (Fig. 1). This shows that diverse raw datasets from SquiDBase can be leveraged to define operating limits and potentially guide parameter selection for upcoming experiments or analyses.

Diverse raw nanopore datasets serve critical functions beyond standard benchmarking, enabling novel signal-level analyses. Two publications with datasets deposited in SquiDBase illustrate complementary signal-level applications: A first study benchmarked cgMLST outbreak analysis for *Salmonella enterica* and *Neisseria meningitidis* using ONT R10.4.1 sequencing and found high locus recovery but basecalling errors for *Neisseria meningitidis* with the rapid barcoding kit (RBK). Raw signal analysis showed that methylation affects these errors [39]. A second study describes a deep-learning framework trained on raw signals to distinguish viable from UV-killed *E. coli* that is transferable to estimate viability in *Chlamydia* but with limits under different killing methods such as antibiotic exposure that required retraining the model [40].

Our roadmap prioritizes expanding microbial R10.4.1 datasets in SquiDBase while improving querying capabilities at the POD5 read level. Key future developments include enhanced metadata visualization, alignment features to facilitate modification analysis, and rapid signal-based searches. As SquiDBase evolves, it will become a more comprehensive platform for storing, retrieving, and analyzing raw nanopore data from microbes. Managing the large file sizes associated with raw nanopore data remains a major challenge. While alternative formats such as SLOW5 exist, we continue to use POD5, the format endorsed by ONT, despite its trade-offs. At present, our focus remains on microbial datasets due to their relatively small genome size, but future developments—driven by technological advancements and community demand—may allow us to broaden the scope to additional organisms and explore more efficient file formats.

In conclusion, SquiDBase addresses the urgent need for a centralized repository of raw nanopore data, promoting open science and enabling the research community to fully leverage nanopore sequencing technology. Worth mentioning is the analogy to the field of proteomics, where the practice of sharing raw mass spectrometry data in public repositories has driven algorithm improvements and novel discoveries [41]. Following this model, we propose that SquiDBase will improve the long-term research utility of nanopore sequencing data, catalyzing breakthroughs in diagnostics, base modification research, and the development of algorithms that can process vast amounts of nanopore data in resource-limited settings. Central to these innovations is the availability of extensive datasets for model training, re-analysis, meta-analyses, and benchmarking.

## Supplementary Material

lqaf213_Supplemental_File

## Data Availability

The data underlying this article are available in SquiDBase at https://squidbase.org/ and can be accessed via their unique submission identifiers (e.g. https://squidbase.org/submissions/SQB000004). Each submission page provides raw signals, full metadata, versioned basecalled outputs, and direct download links.
