# Supplementary material for: SquiDBase: a community resource of raw nanopore data from microbes
Source: NAR Genom Bioinform. 2026 Jan 8;8(1):lqaf213. doi: 10.1093/nargab/lqaf213 (PMC12783041; doi:10.1093/nargab/lqaf213)
Supplement: lqaf213_Supplemental_File [file lqaf213_supplemental_file.docx]

**SUPPLEMENTARY MATERIALS**

**SquiDBase: a community resource of raw nanopore data from microbes**

Wim L. Cuypers^1*^, Halil Ceylan^1^, Eline Turcksin^1^, Laura Raes^1^, Nicky de Vrij^1,2^, Johan Michiels^3^, Sandra Coppens^3^, Tessa de Block^4^, Daan Jansen^4^, Kevin K. Ariën^3,4^, Philippe Selhorst^3^, Koen Vercauteren^5^, Julia M. Gauglitz^1^, Wout Bittremieux^1^, Kris Laukens^1*^

^1^Adrem Data Lab, Department of Computer Science, University of Antwerp

^2^Clinical immunology Unit, Department of Clinical Sciences, Institute of Tropical Medicine Antwerp

^3^Virology Unit, Department of Biomedical sciences, Institute of Tropical Medicine Antwerp

^4^Department of Biomedical Sciences, University of Antwerp

^5^Clinical virology Unit, Department of Clinical Sciences, Institute of Tropical Medicine Antwerp

*Correspondence should be addressed to W.L.C and K.L (email: wim.cuypers@uantwerpen.be and kris.laukens@uantwerpen.be)

**Table of contents**

[Supplementary Methods 3](#_Toc211367971)

[Section 1: The SquiDPipe preprocessing pipeline 3](#_Toc211367972)

[Section 2: Sample processing 4](#_Toc211367973)

[Section 3: Raw signal matching use-case 6](#_Toc211367974)

[Supplementary Tables 7](#_Toc211367975)

[Supplementary Figures 13](#_Toc211367976)

[References 18](#_Toc211367977)

# Supplementary Methods

## Section 1: The SquiDPipe preprocessing pipeline

**For processing microbial/virus isolates and metagenomes**, SquiDPipe (<https://github.com/SquiDBase/SquiDPipe>) accepts demultiplexed POD5 and FASTQ files organized per barcode alongside a sample-sheet CSV mapping barcodes to sample labels and, optionally, NCBI TaxIDs and other SquiDBase-required metadata. Input validation includes filename integrity checks and barcode resolution across FASTQ and POD5 sources. Upon successful validation, the workflow partitions raw signals by barcode using the POD5 package (<https://github.com/nanoporetech/pod5-file-format>) and generates corresponding metadata entries, with optional emission of SquiDBase-compatible CSV outputs. Importantly, for these isolate and metagenome datasets, SquiDPipe operates in a non-classifying mode where POD5 files are subset strictly by barcode. FASTQ read identifiers are used to locate matching signal-level entries in the POD5 files, ensuring lossless splitting without downstream taxonomic screening or reference alignment. This mode is optimized for speed and minimal transformation of source signals. Parameter defaults and CSV schema definitions are available in the SquiDPipe repository README. The output directory includes: (*i*) POD5 files per barcode renamed according to species name abbreviation and taxonomic identifier, and (*ii*) the formatted metadata CSV (if requested) matching the new filenames.

**For removing human host reads in clinical samples**, SquiDPipe performs taxonomic screening followed by competitive alignment to remove host sequences prior to extracting target signals (e.g., microbe, virus). Inputs mirror those of isolate-mode runs, with two additional required parameters: (*i*) a host reference genome (e.g., GRCh38) for alignment, and (*ii*) an NCBI taxonomic identifier specifying the microbial target(s) when per-taxon outputs are desired. Reads are first classified with Kraken2 against a user-selected database (e.g., RefSeq Virus). Reads assigned to the specified microbial target are extracted and subjected to competitive read mapping with minimap2 using a composite reference comprising the host “decoy” genome and the target genome. Target references are retrieved automatically using ncbi-datasets: SquiDPipe queries RefSeq first, falls back to GenBank if unavailable, and, if no species-level match is found, ascends the taxonomy to select the most complete higher-rank reference (e.g., genus) available. The selected target and decoy sequences are concatenated into a single FASTA for alignment. Following competitive mapping, reads that preferentially align to the host, and potential false-positive target assignments, are removed. The remaining target-assigned signals are written to per-taxon POD5 files. The pipeline also reports mapping statistics to facilitate quality assessment.

SquiDPipe is implemented in Nextflow (DSL2)^1^ and supports containerized and native deployments via Docker, Apptainer or Conda. All software dependencies are pinned and version-controlled in the repository’s environment specifications to ensure reproducibility. The workflow bundles the following tools: Kraken2^2^, minimap2^3^, samtools^4,5^, ncbi-datasets-cli^6^, seqtk^7^, pigz, and pod5. Up-to-date environment definitions and run instructions are provided in the GitHub repository. The README “Usage” section includes command-line examples for host-read removal, and exemplar parameter files. Guidance for packaging outputs for upload is available in the SquiDBase documentation (https://docs.squidbase.org/).

## Section 2: Sample processing

Virus samples were processed by the Clinical Virology and Virology Units at the Institute of Tropical Medicine Antwerp (ITM). For monkeypox virus, DNA was extracted and amplified using sequence-independent single-primer amplification (SISPA), following the protocol described by De Baetselier et al. (2022)^8^. For RNA viruses, the starting material consisted of culture supernatant obtained from the Virology Unit virus repository. The extraction and enrichment protocol included RNA extraction, TurboDNase treatment, reverse transcription to cDNA, and subsequent SISPA amplification, in line with previous publications by Greninger et al. (2015)^9^ and Kafetzopoulou et al. (2018)^10^. Sequencing of the viral strains was carried out at the University of Antwerp using the ONT MinION platform on R10.4.1 flow cells (FLO-MIN114). Library preparation was performed using the Rapid Barcoding Kit v14 (SQK-RBK114.24), and barcoded libraries were sequenced in batches of 24 pooled samples per flow cell for 42 to 72 hours. The output ranged from 1.88 up to 5.22 gigabase. Raw nanopore reads in POD5 format were basecalled using Dorado (ONT Dorado basecall server software version 7.0.2+7e7b7d0) employing the super-accurate (SUP) model (dna_r10.4.1_e8.2_400bps_5khz). Data were further processed using SquiDPipe v1.0.0 (https://github.com/SquiDBase/SquiDPipe), implemented in Nextflow v24.04.4^1^. In brief, reads were classified and extracted using Kraken2 v2.1.3^2^ with the RefSeq viral database^11^ (downloaded May 25, 2024) and Seqtk v1.4-r122^7^. Reference genomes for target species and human (to account for contamination) were retrieved using the NCBI datasets CLI v16.27.1^6^. Reads were aligned to concatenated reference genomes using Minimap2 v2.28-r1209^3^, and alignments were processed with Samtools v1.20^4,5^ to remove duplicates, generate BAM files, and calculate coverage statistics. Finally, the Pod5 package v0.3.15^12^ was used to extract reads mapping exclusively to the target species, consolidating them into single POD5 files per species.

To further expand the pathogen diversity represented in SquiDBase, we incorporated raw nanopore whole-genome sequencing data from Plasmodium falciparum, a clinically significant pathogen for which such data were previously unavailable. The complete wet lab protocol is available in the study by De Meulenaere et al. (2024)^13^. We ran SquiDPipe v1.0.0 to extract raw nanopore signal data specific to Plasmodium. Using this approach, we obtained data for Plasmodium falciparum from three human patients, with native parasite DNA sequenced directly from blood using nanopore sequencing in adaptive sampling mode. For one of these samples, we also generated sWGA-enriched data in addition to the native DNA dataset.

## Section 3: Raw signal matching use-case

Data were downloaded from SquiDBase, using the ‘Download All’ feature, that generates curl links that allow downloading the entire submission or subsets thereof directly to a local machine. Details on the datasets, reference genomes used for alignment and basecalling specifications are listed in **Supplementary Table 4**.

RawHash2 v2.1 was executed in sensitive mode, which is the parameter preset recommended for reference genomes below 500 Mb. For R9 data, the template_median68pA.model was used that is packaged with the tool. For R10 data, the R10 preset was used, using the pore model ‘uncalled_r1041_model_only_means.txt’ from UNCALLED4, as recommended by the tool authors^14,15^. Unless noted otherwise, all other parameters were left at their documented defaults. Ground-truth labels were assigned from ONT “SUP” basecalled reads aligned to their corresponding reference genome with minimap2 v2.30-r1287^3^. Classification accuracy was defined at the read level as the proportion of all input reads whose tool-reported primary assignment matched the ground-truth reference genome; unmapped or ambiguously assigned reads were counted as incorrect. Pairwise comparisons and summary statistics were computed from PAF outputs using pafstats v2.3. All steps were combined in a reproducible workflow using Nextflow v25.04.2^1^.

# Supplementary Tables

**Supplementary Table 1**: Description of the PostgreSQL database tables in SquiDBase.

| Table name | Description |
| --- | --- |
| users | Stores user information. Essential for managing user accounts and linking them to submissions. |
| country | Holds country data using ISO codes. Used to reference geographic locations in other tables. |
| nanopore_chemistry | Lists types of nanopore chemistries. |
| nanopore_kit | Contains details about various nanopore kits. |
| source | Stores source information from various ontologies (UBERON, FOODON, ENVO). Provides standardized references. |
| submissions | Manages data submission records. |
| ncbi_taxon | Contains taxonomic data with hierarchical relationships, essential for biological classification. |
| pod5_file | Represents files with metadata linking to countries, taxonomies, and submissions for comprehensive tracking. |
| pod5_read | Stores read-level data linked to files. Ensures each read is associated with a valid file. |
| diagnostic | Stores source information from OBI ontology. Provides standardized references. |
| alembic_version | Maintains a record of applied database schema migrations, ensuring version control and reproducibility across development environments. It provides standardized tracking of structural changes to the database. |

**Supplementary Table 2**: Coverage statistics for virus isolates in SquiDBase. The table includes the following columns: "Name" (virus abbreviation, e.g., DENV1), "StudyID" (internal lab identifier), “NCBI TaxID” (taxonomic identifier), "Chromosome" (RefSeq identifier of the reference genome used for read mapping), "Coverage" (proportion of the reference genome covered by at least one read), "Depth of Coverage" (average number of reads mapped to a position in the reference genome). The remaining columns indicate the percentage of positions in the reference genome covered by at least 10, 20, or 30 reads respectively.

| Name | studyID | NCBI TaxID | Chromosome | Coverage (%) | Depth of Coverage | Positions  > 10  reads (%) | Positions > 20  reads (%) | Positions > 30  reads (%) |
| --- | --- | --- | --- | --- | --- | --- | --- | --- |
| CHIKV | 1 | 37124 | NC_004162.2 | 100.0 | 486.1 | 98.4 | 97.6 | 96.6 |
| CHIKV | 2 | 37124 | NC_004162.2 | 100.0 | 739.9 | 99.7 | 99.1 | 98.6 |
| CHIKV | 3 | 37124 | NC_004162.2 | 99.87 | 299.8 | 99.1 | 98.5 | 97.6 |
| CHIKV | 4 | 37124 | NC_004162.2 | 99.94 | 75.3 | 82.3 | 76.3 | 70.3 |
| DENV1 | 5 | 11053 | NC_001477.1 | 99.96 | 602.5 | 98.9 | 98.3 | 98.2 |
| DENV1 | 6 | 11053 | NC_001477.1 | 99.59 | 764.8 | 99.0 | 98.9 | 98.8 |
| DENV2 | 7 | 11060 | NC_001474.2 | 99.68 | 531.7 | 98.8 | 98.3 | 97.9 |
| DENV2 | 8 | 11060 | NC_001474.2 | 99.67 | 973.4 | 99.1 | 99.1 | 98.9 |
| DENV2 | 9 | 11060 | NC_001474.2 | 99.42 | 208.3 | 98.2 | 96.6 | 96.1 |
| DENV3 | 10 | 11069 | NC_001475.2 | 99.05 | 232.0 | 98.8 | 98.0 | 97.6 |
| DENV3 | 11 | 11069 | NC_001475.2 | 99.97 | 772.4 | 99.1 | 98.9 | 98.8 |
| DENV3 | 12 | 11069 | NC_001475.2 | 99.84 | 172.8 | 98.3 | 97.3 | 95.9 |
| DENV3 | 68 | 11069 | NC_001475.2 | 99.69 | 685.0 | 99.1 | 99.1 | 98.9 |
| DENV4 | 13 | 11070 | NC_002640.1 | 99.97 | 667.4 | 99.4 | 99.1 | 98.7 |
| DENV4 | 14 | 11070 | NC_002640.1 | 99.94 | 173.8 | 98.1 | 97.4 | 96.9 |
| DENV4 | 15 | 11070 | NC_002640.1 | 99.68 | 227.3 | 98.5 | 97.5 | 97.4 |
| EEEV | 16 | 11021 | NC_003899.1 | 99.99 | 544.5 | 99.7 | 99.6 | 99.5 |
| HIV | 59 | 11676 | NC_001802.1 | 100.0 | 332.2 | 97.2 | 96.7 | 96.2 |
| HIV | 60 | 11676 | NC_001802.1 | 99.93 | 299.5 | 96.7 | 93.7 | 92.3 |
| HIV | 61 | 11676 | NC_001802.1 | 99.93 | 552.7 | 96.9 | 95.2 | 93.8 |
| HIV | 62 | 11676 | NC_001802.1 | 99.98 | 414.3 | 97.4 | 96.4 | 95.7 |
| HIV | 63 | 11676 | NC_001802.1 | 99.84 | 221.6 | 96.2 | 93.4 | 90.3 |
| HIV | 64 | 11676 | NC_001802.1 | 97.79 | 193.00 | 93.1 | 88.1 | 85.7 |
| JEV | 17 | 11072 | NC_001437.1 | 99.99 | 508.8 | 99.1 | 99.0 | 99.0 |
| JEV | 18 | 11072 | NC_001437.1 | 100.0 | 763.8 | 99.3 | 99.1 | 99.0 |
| JEV | 19 | 11072 | NC_001437.1 | 99.45 | 198.5 | 99.0 | 98.9 | 98.8 |
| JEV | 20 | 11072 | NC_001437.1 | 99.13 | 199.5 | 99.0 | 98.8 | 98.8 |
| MAYV | 21 | 59301 | NC_003417.1 | 98.09 | 215.8 | 97.8 | 97.6 | 94.5 |
| MPOX | 65 | 10244 | NC_063383.1 | 45.67 | 0.7 | 0 | 0 | 0 |
| MPOX | 66 | 10244 | NC_063383.1 | 9.73 | 0.1 | 0 | 0 | 0 |
| MPOX | 67 | 10244 | NC_063383.1 | 99.06 | 14.5 | 69.0 | 21.1 | 6.1 |
| ONNV | 22 | 2169701 | NC_001512.1 | 99.97 | 415.2 | 98.8 | 97.9 | 97.1 |

**Supplementary Table 2** continued

| Name | studyID | NCBI TaxID | Chromosome | Coverage (%) | Depth of Coverage | Positions  > 10  reads (%) | Positions > 20  reads (%) | Positions > 30  reads (%) |
| --- | --- | --- | --- | --- | --- | --- | --- | --- |
| RRV | 23 | 11029 | NC_075016.1 | 100.0 | 813.1 | 99.7 | 99.6 | 99.5 |
| RVFV | 24 | 11588 | NC_014395.1 | 100.0 | 168.6 | 100.0 | 99.8 | 90.2 |
| RVFV | 24 | 11588 | NC_014396.1 | 100.0 | 270.1 | 100.0 | 99.1 | 98.6 |
| RVFV | 24 | 11588 | NC_014397.1 | 100.0 | 298.7 | 100.0 | 100.0 | 100.0 |
| SARS-CoV-2 | 25 | 2697049 | NC_045512.2 | 100.0 | 400.2 | 99.9 | 99.8 | 99.8 |
| SARS-CoV-2 | 26 | 2697049 | NC_045512.2 | 99.9 | 294.0 | 99.8 | 99.7 | 99.7 |
| SARS-CoV-2 | 27 | 2697049 | NC_045512.2 | 100.0 | 276.0 | 99.8 | 99.7 | 99.7 |
| SARS-CoV-2 | 28 | 2697049 | NC_045512.2 | 100.0 | 520.7 | 99.9 | 99.8 | 99.7 |
| SARS-CoV-2 | 29 | 2697049 | NC_045512.2 | 99.9 | 165.6 | 99.6 | 99.5 | 99.5 |
| SARS-CoV-2 | 30 | 2697049 | NC_045512.2 | 99.9 | 330.8 | 99.7 | 99.6 | 99.6 |
| SARS-CoV-2 | 31 | 2697049 | NC_045512.2 | 99.9 | 95.2 | 99.8 | 99.5 | 99.3 |
| SARS-CoV-2 | 32 | 2697049 | NC_045512.2 | 99.9 | 77.9 | 99.7 | 99.4 | 97.2 |
| SARS-CoV-2 | 69 | 2697049 | NC_045512.2 | 100.0 | 388.7 | 99.8 | 99.8 | 99.8 |
| SARS-CoV-2 | 70 | 2697049 | NC_045512.2 | 99.8 | 96.8 | 99.6 | 99.4 | 98.5 |
| SFNV | 35 | 206160 | NC_078077.1 | 100.0 | 184.3 | 99.8 | 98.8 | 98.1 |
| SFNV | 35 | 206160 | NC_078078.1 | 100.0 | 182.1 | 99.8 | 98.9 | 98.0 |
| SFNV | 35 | 206160 | NC_078079.1 | 100.0 | 274.2 | 99.4 | 97.3 | 95.8 |
| SFNV | 42 | 206160 | NC_078077.1 | 25.9 | 1.9 | 5.2 | 2.5 | 2.2 |
| SFNV | 42 | 206160 | NC_078078.1 | 0 | 0 | 0 | 0 | 0 |
| SFNV | 42 | 206160 | NC_078079.1 | 36.2 | 0.8 | 0 | 0 | 0 |
| SFTV | 36 | 688699 | NC_015411.1 | 99.8 | 214.4 | 96.9 | 93.4 | 92.3 |
| SFTV | 36 | 688699 | NC_015412.1 | 100.0 | 222.2 | 99.5 | 98.3 | 96.8 |
| SFTV | 36 | 688699 | NC_015413.1 | 100.0 | 573.4 | 100.0 | 100.0 | 99.8 |
| SINV | 37 | 11034 | NC_001547.1 | 100.0 | 411.9 | 99.8 | 99.5 | 98.5 |
| TBEV | 38 | 11084 | NC_001672.1 | 97.3 | 608.5 | 96.1 | 95.4 | 94.5 |
| TBEV | 39 | 11084 | NC_001672.1 | 97.2 | 524.0 | 95.3 | 93.9 | 93.9 |
| TBEV | 40 | 11084 | NC_001672.1 | 96.8 | 159.6 | 93.9 | 93.8 | 93.7 |
| TBEV | 41 | 11084 | NC_001672.1 | 98.4 | 132.0 | 96.8 | 96.2 | 95.4 |
| USUV | 43 | 64286 | NC_006551.1 | 100.0 | 467.9 | 99.4 | 99.0 | 98.9 |
| USUV | 44 | 64286 | NC_006551.1 | 100.0 | 867.5 | 99.8 | 99.4 | 99.0 |
| VEEV | 46 | 11036 | NC_075022.1 | 100.0 | 957.6 | 99.8 | 99.7 | 99.6 |
| WEEV | 47 | 11039 | NC_075015.1 | 98.6 | 550.2 | 98.3 | 98.3 | 98.3 |
| WNV | 48 | 11082 | NC_009942.1 | 100.0 | 639.2 | 99.9 | 99.3 | 99.2 |
| WNV | 49 | 11082 | NC_009942.1 | 100.0 | 432.8 | 99.2 | 99.1 | 99.1 |
| WNV | 50 | 11082 | NC_009942.1 | 60.4 | 9.28 | 23.0 | 14.3 | 11.9 |
| WNV | 51 | 11082 | NC_009942.1 | 77.2 | 17.6 | 42.3 | 27.1 | 18.6 |

**Supplementary Table 2** continued

| Name | studyID | NCBI TaxID | Chromosome | Coverage (%) | Depth of Coverage | Positions  > 10  reads (%) | Positions > 20  reads (%) | Positions > 30  reads (%) |
| --- | --- | --- | --- | --- | --- | --- | --- | --- |
| YFV | 52 | 11089 | NC_002031.1 | 99.9 | 415.3 | 99.3 | 99.0 | 99.0 |
| YFV | 53 | 11089 | NC_002031.1 | 100.0 | 691.7 | 99.9 | 99.2 | 99.1 |
| YFV | 54 | 11089 | NC_002031.1 | 99.9 | 184.2 | 99.0 | 98.9 | 98.9 |
| ZIKV | 55 | 64320 | NC_035889.1 | 99.9 | 228.6 | 98.9 | 98.8 | 98.8 |
| ZIKV | 56 | 64320 | NC_035889.1 | 99.5 | 377.4 | 98.9 | 98.8 | 98.4 |
| ZIKV | 57 | 64320 | NC_035889.1 | 99.0 | 173.7 | 98.7 | 98.6 | 97.4 |
| ZIKV | 58 | 64320 | NC_035889.1 | 99.6 | 192.2 | 98.7 | 97.7 | 96.9 |

**Supplementary Table 3**: Coverage statistics for clinical Plasmodium samples in SquiDBase. For each of the three patient samples (PS1, PS2, and PS3), depth and coverage are presented relative to the Plasmodium falciparum 3D7 reference genome. Further details on the sequencing methodology can be found in De Meulenaere et al. (2024)^13^.

| Region | PS1 Mean depth | PS1 Coverage (%) | PS2 Mean depth | PS2 Coverage (%) | PS3 Mean depth | PS3 Coverage (%) | PS3 Mean depth | PS3 Coverage (%) |
| --- | --- | --- | --- | --- | --- | --- | --- | --- |
| full genome | 19.7 | 99.8 | 5.6 | 97.4 | 351.5 | 99.9 | 99.9 | 335.1 |
| chr 1 | 19.6 | 99.5 | 6.1 | 97.0 | 335.9 | 99.8 | 99.8 | 332.0 |
| chr 2 | 19.9 | 99.8 | 5.2 | 97.2 | 356.6 | 100.0 | 100.0 | 344.1 |
| chr 3 | 20.2 | 99.9 | 6.0 | 97.4 | 359.3 | 100.0 | 100.0 | 357.0 |
| chr 4 | 19.1 | 99.5 | 5.3 | 94.7 | 371.2 | 99.9 | 99.9 | 348.9 |
| chr 5 | 19.2 | 99.9 | 5.2 | 98.2 | 340.5 | 99.9 | 99.9 | 324.9 |
| chr 6 | 19.7 | 99.6 | 5.5 | 96.9 | 346.6 | 99.7 | 99.8 | 340.1 |
| chr 7 | 18.4 | 99.1 | 5.6 | 95.0 | 341.8 | 99.6 | 99.9 | 328.9 |
| chr 8 | 19.9 | 99.9 | 5.7 | 95.7 | 334.8 | 99.7 | 99.8 | 316.3 |
| chr 9 | 19.6 | 99.9 | 5.5 | 98.1 | 354.3 | 99.9 | 100.0 | 340.8 |
| chr 10 | 19.4 | 99.9 | 5.4 | 97.8 | 339.6 | 100.0 | 100.0 | 323.0 |
| chr 11 | 20.2 | 99.8 | 5.5 | 97.9 | 350.6 | 99.9 | 100.0 | 325.5 |
| chr 12 | 19.0 | 99.8 | 5.9 | 96.8 | 344.1 | 99.9 | 100.0 | 327.8 |
| chr 13 | 20.3 | 99.9 | 5.5 | 98.6 | 360.0 | 100.0 | 100.0 | 349.4 |
| chr 14 | 19.6 | 99.9 | 5.7 | 98.4 | 349.6 | 100.0 | 100.0 | 332.6 |

**Supplementary Table 4:** Datasets used to benchmark RawHash2 and hosted on SquiDBase. Each row lists the SquiDBase accession, organism/strain, ONT flow cell version (R9.4.1, R10.4, R10.4.1), source study (year), the reference genome or isolate identifier used for alignment, and the basecalling software with model name/version used to generate the associated FASTQ files.

| SquiDBase ID | Organism (strain) | ONT chemistry | Study | Reference genome / ID | Basecaller + version | Basecalling model |
| --- | --- | --- | --- | --- | --- | --- |
| SQB000001 | *Escherichia* *coli* CFT073 | R9.4.1 | Sanderson et al. (2023)^16^ | GCA_000007445.1 | Dorado v0.9.1 | dna_r9.4.1_e8_sup@v3.6 |
| SQB000012 | *Escherichia* *coli* CFT073 | R10.4 | Sanderson et al. (2023) ^16^ | GCA_000007445.1 | Guppy v6.5.7 | dna_r10.4_e8.1_sup.cfg |
| SQB000011 | *Escherichia* *coli* ATCC 25922 | R10.4.1 | Hall et al. (2024)^17^ | GCA_000743255.1 | Dorado v0.5.0 | dna_r10.4.1_e8.2_400bps_sup@v4.3.0 |
| SQB000013 | *Salmonella* *enterica* serovar Concord | R9.4.1 | Cuypers et al. (2023)^18^ | 32640_1_47 | Dorado v0.9.1 | dna_r9.4.1_e8_sup@v3.6 |
| SQB000011 | *Salmonella* *enterica* serovar Choleraesuis strain ETS 34 | R10.4.1 | Hall et al. (2024)^17^ | ATCC_10708__202309 | Dorado v0.5.0 | dna_r10.4.1_e8.2_400bps_sup@v4.3.0 |

# Supplementary Figures


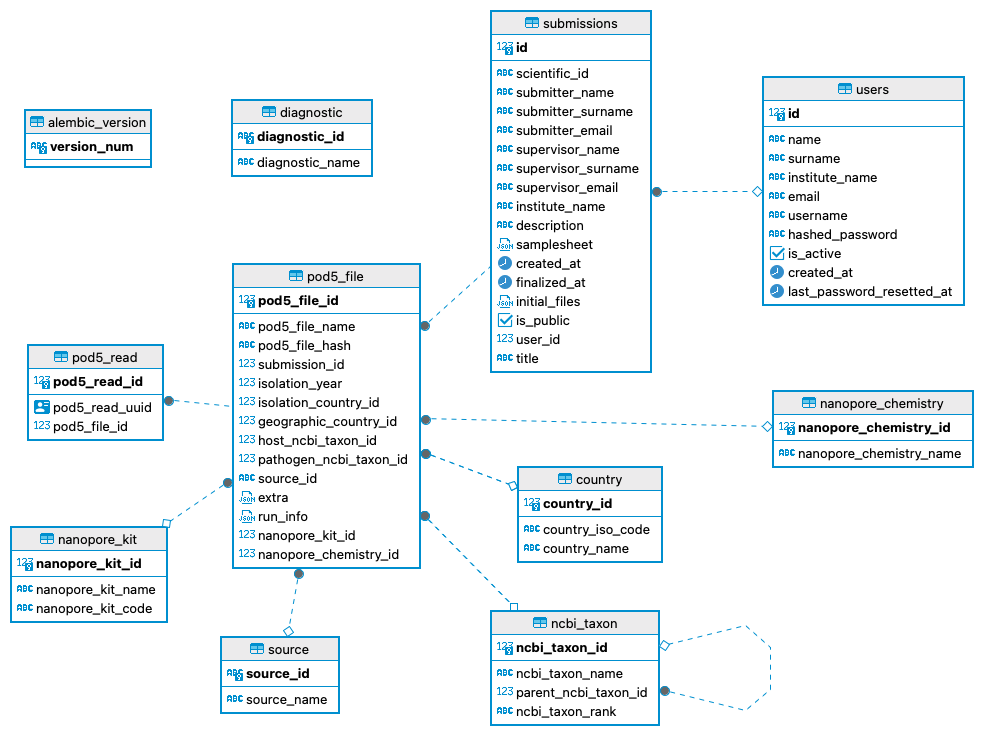


**Supplementary Figure 1**: Structure of the SQL part of SquiDBase.


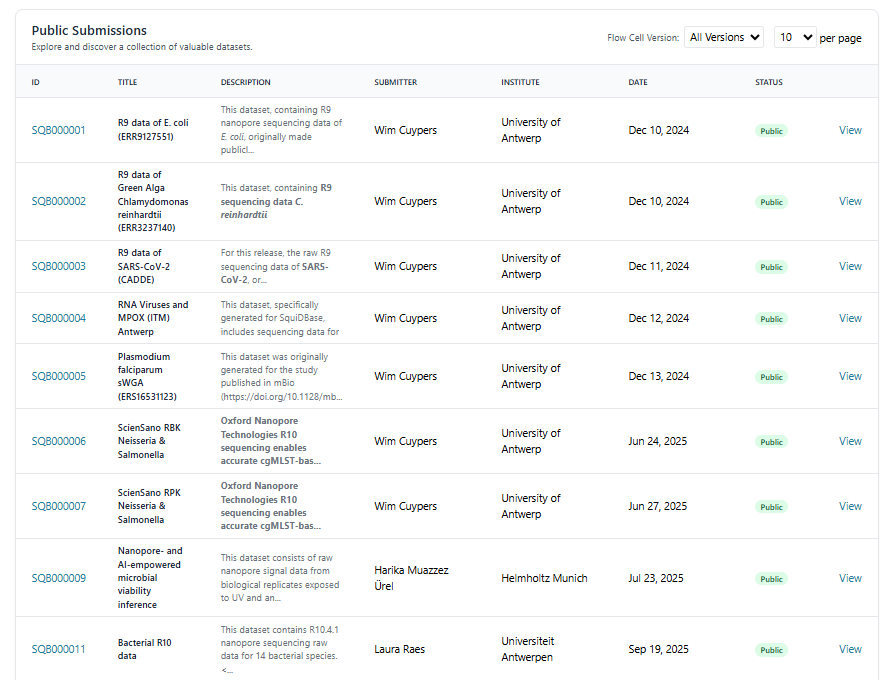


**Supplementary Figure 2**: The ‘Browse Datasets’ page in SquiDBase displays each dataset with a unique SQB identifier, title, description, and details about the submitter (name, institute) along with the submission date. This page is accessible at <https://squidbase.org/submissions>.


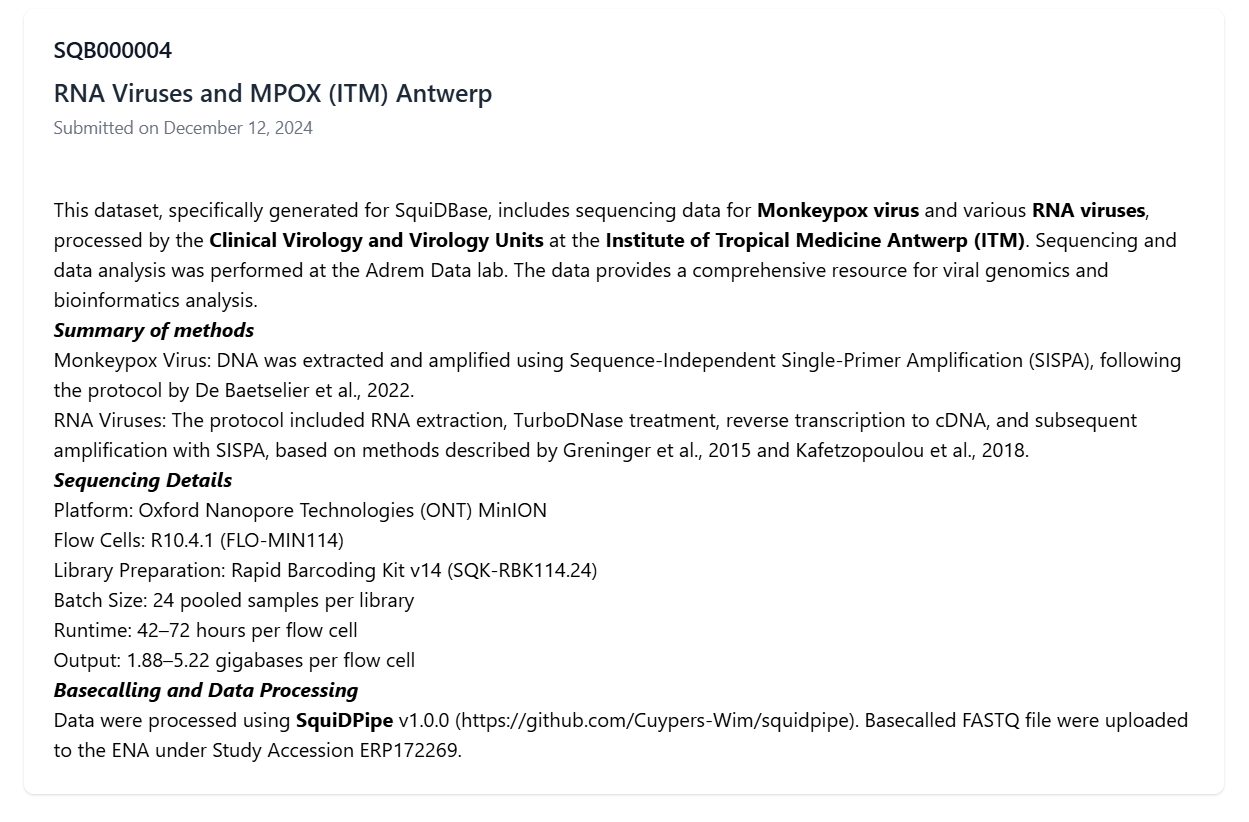


**
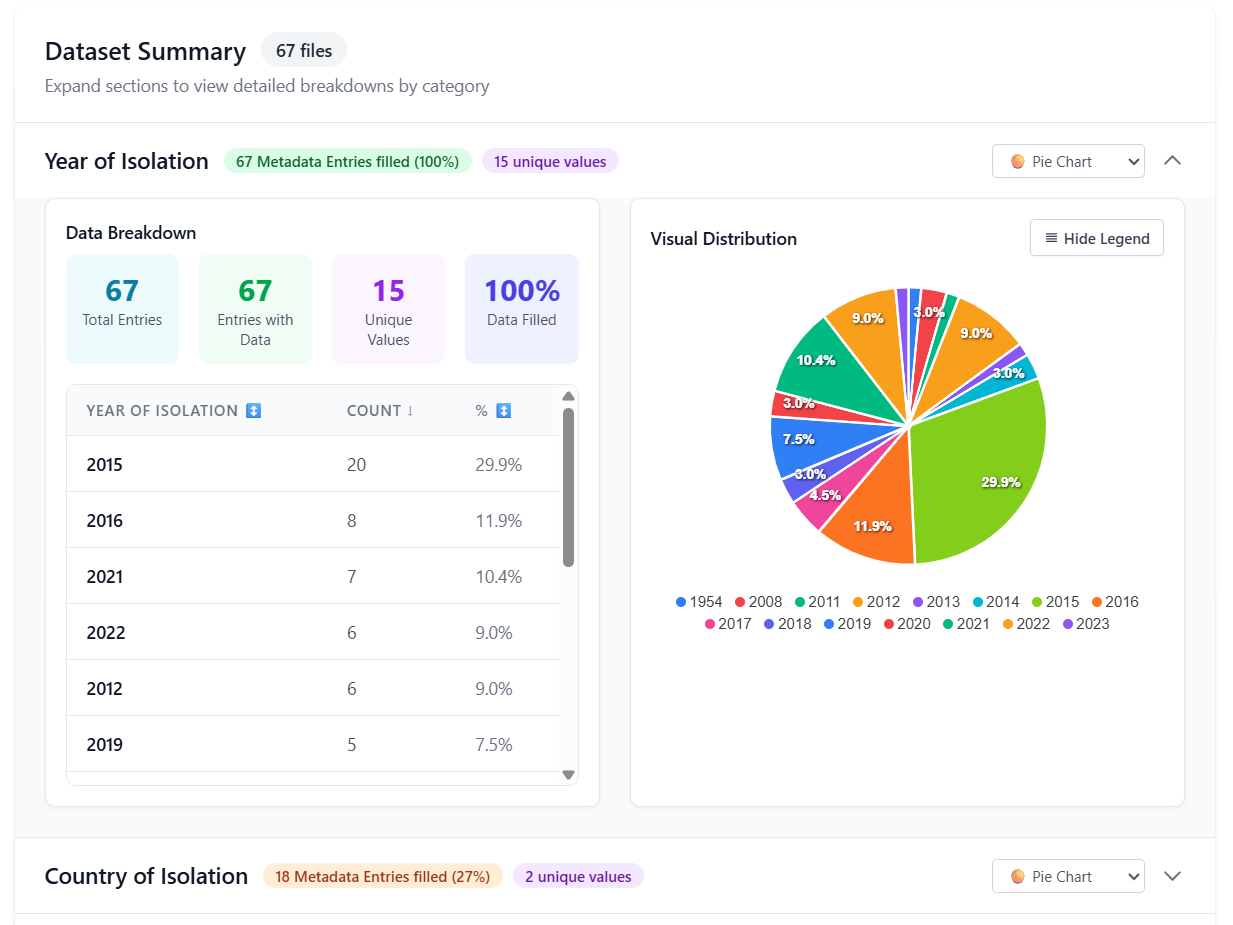
**


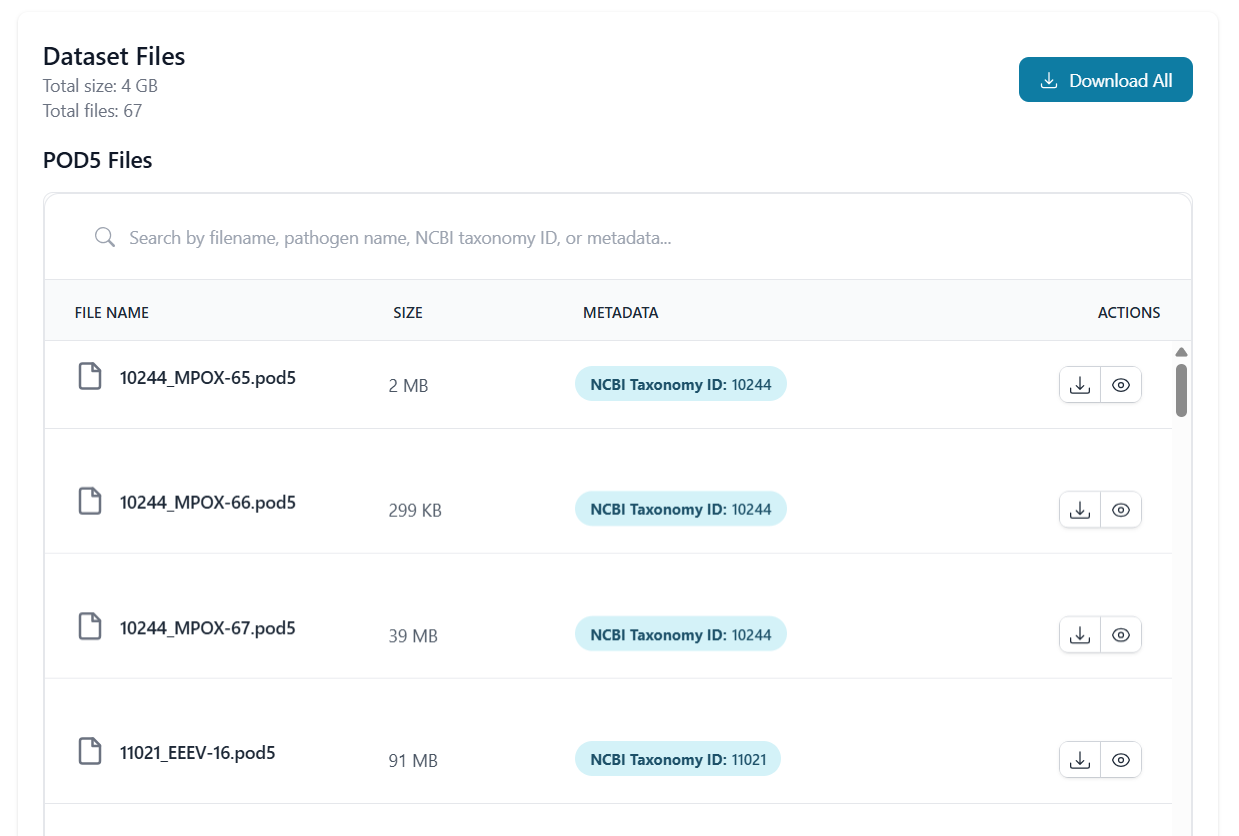


**Supplementary Figure 3**: Dataset entry linked to a SquiDBase identifier. The page shows a textual summary of the dataset, followed by a visual summary of the available metadata followed by a ‘Dataset Files’ screen to download all data files separately. This screenshot was generated from <https://squidbase.org/submissions/SQB000004>.


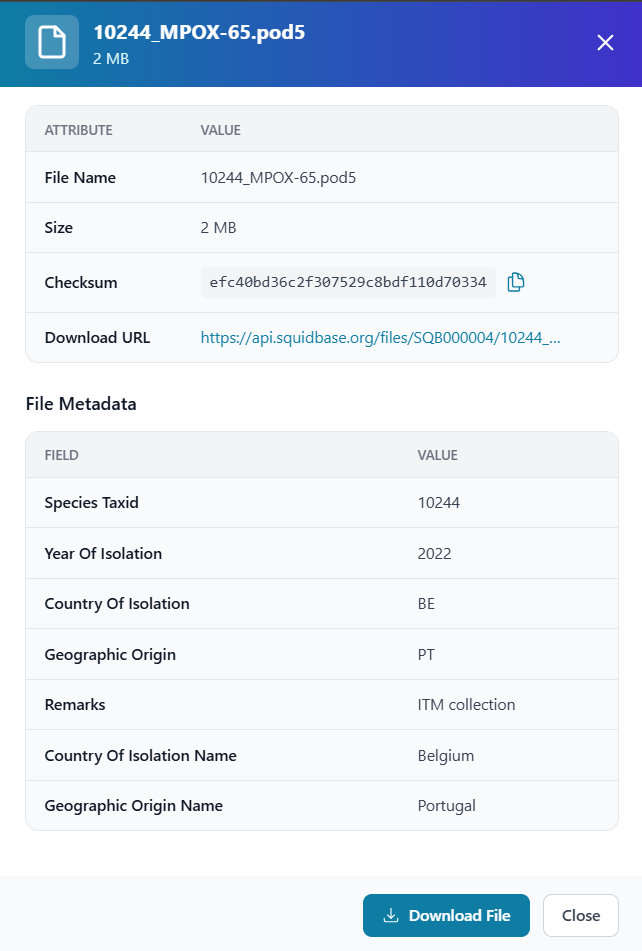


**Supplementary Figure 4**: In the 'Dataset Files' section for each dataset submission, the eye icon expands to reveal complete metadata, including an MD5 checksum for file integrity verification.

# References

1. Di Tommaso, P. *et al.* Nextflow enables reproducible computational workflows. *Nat. Biotechnol.* **35**, 316–319 (2017).

2. Wood, D. E., Lu, J. & Langmead, B. Improved metagenomic analysis with Kraken 2. *Genome Biol.* **20**, 257 (2019).

3. Li, H. Minimap2: pairwise alignment for nucleotide sequences. *Bioinformatics* **34**, 3094–3100 (2018).

4. Li, H. *et al.* The Sequence Alignment/Map format and SAMtools. *Bioinformatics* **25**, 2078–2079 (2009).

5. Danecek, P. *et al.* Twelve years of SAMtools and BCFtools. *Gigascience* **10**, (2021).

6. O’Leary, N. A. *et al.* Exploring and retrieving sequence and metadata for species across the tree of life with NCBI Datasets. *Sci. Data* **11**, 732 (2024).

7. Li, H. *Seqtk: Toolkit for Processing Sequences in FASTA/Q Formats*. (Github).

8. De Baetselier, I. *et al.* Retrospective detection of asymptomatic monkeypox virus infections among male sexual health clinic attendees in Belgium. *Nat. Med.* **28**, 2288–2292 (2022).

9. Greninger, A. L. *et al.* Rapid metagenomic identification of viral pathogens in clinical samples by real-time nanopore sequencing analysis. *Genome Med.* **7**, 99 (2015).

10. Kafetzopoulou, L. E. *et al.* Assessment of metagenomic Nanopore and Illumina sequencing for recovering whole genome sequences of chikungunya and dengue viruses directly from clinical samples. *Euro Surveill.* **23**, (2018).

11. O’Leary, N. A. *et al.* Reference sequence (RefSeq) database at NCBI: current status, taxonomic expansion, and functional annotation. *Nucleic Acids Res.* **44**, D733-45 (2016).

12. *Pod5-File-Format: Pod5: A High Performance File Format for Nanopore Reads*. (Github).

13. De Meulenaere, K. *et al.* Selective whole-genome sequencing of *Plasmodium* parasites directly from blood samples by nanopore adaptive sampling. *MBio* **15**, (2024).

14. Firtina, C., Soysal, M., Lindegger, J. & Mutlu, O. RawHash2: mapping raw nanopore signals using hash-based seeding and adaptive quantization. *Bioinformatics* **40**, (2024).

15. Kovaka, S. *et al.* Uncalled4 improves nanopore DNA and RNA modification detection via fast and accurate signal alignment. *Nat. Methods* **22**, 681–691 (2025).

16. Sanderson, N. D. *et al.* Comparison of R9.4.1/Kit10 and R10/Kit12 Oxford Nanopore flowcells and chemistries in bacterial genome reconstruction. *Microb. Genom.* **9**, (2023).

17. Hall, M. B. *et al.* Benchmarking reveals superiority of deep learning variant callers on bacterial nanopore sequence data. *Elife* **13**, (2024).

18. Cuypers, W. L. *et al.* A global genomic analysis of Salmonella Concord reveals lineages with high antimicrobial resistance in Ethiopia. *Nat. Commun.* **14**, 3517 (2023).
